# Global Research on Smoking and Pregnancy—A Scientometric and Gender Analysis

**DOI:** 10.3390/ijerph110605792

**Published:** 2014-05-28

**Authors:** Mathias Mund, Beatrix Kloft, Matthias Bundschuh, Doris Klingelhoefer, David A. Groneberg, Alexander Gerber

**Affiliations:** 1 Institute of Occupational Medicine, Social Medicine and Environmental Medicine, Goethe University, Frankfurt am Main, Theodor-Stern-Kai 7, Frankfurt 60590, Germany; E-Mails: M-coder@hotmail.com (M.M.); Bundschuh@med.uni-frankfurt.de (M.B.); arbsozmed@uni-frankfurt.de (D.A.G.); gerber@med.uni-frankfurt.de (A.G.); 2 Department of Obstetrics and Gynaecology, Goethe University, Frankfurt am Main, Theodor-Stern-Kai 7, Frankfurt 60590, Germany; E-Mail: Beakloft@gmx.de

**Keywords:** bibliometry, cigarette, citation, gender studies, pregnancy, scientometry, smoking

## Abstract

The exposure to tobacco smoke during pregnancy is considered to be amongst the most harmful avoidable risk factors. In this scientometric and gender study scientific data on *smoking and pregnancy* was analyzed using a variety of objective scientometric methods like the number of scientific contributions, the number of citations and the modified h-index in combination with gender-specific investigations. Covering a time period from 1900 to 2012, publishing activities of 27,955 authors, institutions and countries, reception within the international scientific community and its reactions were analyzed and interpreted. Out of 10,043 publications the highest number of scientific works were published in the USA (35.5%), followed by the UK (9.9%) and Canada (5.3%). These nations also achieve the highest modified *h-indices* of 128, 79 and 62 and the highest citation rates of 41.4%, 8.6% and 5.3%, respectively. Out of 12,596 scientists 6,935 are female (55.1%), however they account for no more than 49.7% of publications (12,470) and 42.8% of citations (172,733). The highest percentage of female experts about *smoking and pregnancy* is found in Australasia (60.7%), while the lowest is found in Asia (41.9%). The findings of the study indicate an increase in gender equality as well as in quantity and quality of international scientific research about *smoking and pregnancy* in the future.

## 1. Introduction

It has been proven in many studies that smoking tobacco throughout pregnancy is one of the single most important avoidable causes of adverse pregnancy outcomes, resulting in severe short and long-term negative effects for the mother and the unborn child [1,2,3,4]. However, a detailed gender-specific scientometric investigation about *smoking and pregnancy* has not been published. The aim of this study is to close this gap by analyzing and evaluating scientific data on *smoking and pregnancy.* Additionally, the investigation of the gender of the authors in connection with their productivity and reception within the international scientific community is in the focus of the study, since there has been repeatedly stated an under-representation of female authors in scientific works [5,6,7].

If compared with other risk factors in the perinatal period, exposure to tobacco smoke is considered to be amongst the most harmful [4]. The byproducts of combustion are believed to inflict more damage on the fetus than the nicotine itself, but due to the complexity and number of dangerous substances it is unknown which toxic effect is caused by exactly which product [8]. This is especially significant as the majority of the smoking-induced harm for the unborn fetus is permanent. Even today, modern medicine offers very little or no therapeutic treatments for the long-term negative consequences of being exposed to smoke *in-utero* [9]. The complete cessation of smoking during pregnancy includes numerous health benefits for both the future mother and her offspring [10]. In recent years *smoking and pregnancy* and its different negative effects on the health of mother and child have become the focus of numerous scientific works. This illustrates the current importance of this topic as a research area.

*Scientometry* (literally: *measurement of science*) is a scientific field examining the development, structure, productivity and interconnectivity of scientific work. Quantitative and qualitative analyses are performed by means of scientometric instruments [11]. Today bibliometric analyses grow in significance, as funding for many scientific projects is connected to their respective bibliometric achievement. The significance of scientometric analyses of scientific activity and productivity is undeniable, as funding and therefore indirectly the very existence of institutions are often linked to their scientific output and performance. Important financial support for costly scientific projects is often only provided to those institutions which manage to return the most scientific value for the invested funding [12].

## 2. Experimental Section

A collective volume of 10,043 entries about *smoking and pregnancy* and the corresponding bibliographic data covering a time period from 1900 to December 2012 was obtained from the Thomson Reuters Web of Science (WoS) data base, which is the only multidisciplinary database in the field of medical science which provides bibliographic data in combination with citations. 

In order to arrive as closely as possible at capturing all of the available scientometric data the combination of the termini tobac* and pregnanc* or cigar* and pregnanc* or smok* and pregnanc* was used in the search field. The asterisk (*) was used as a wildcard and enabled the search of any letters in its place. All types of publications were retrieved, since the aim was to provide a survey of the entire scientific output. 

The scientific works about *smoking and pregnancy* were analyzed according to year of publication, number of citations per year, language and form of publication. The bibliography and its development over time as well as the average citation rate per publication was calculated and assessed. The countries of origin were identified and assigned to the respective publications. National modified *h-indices* as well as national citation rates were calculated for each country. 

The authors contributing publications about *smoking and pregnancy* were investigated to determine the most productive and most cited scientists. The average citation rate and modified *h-indices* of the most productive specialists were calculated and compared. A modified h-index based on the original *h-index* was used in this work to exclusively evaluate their scientific importance about *smoking and pregnancy*. The data was further analyzed to evaluate the different types of authorships and the authors’ gender. Out of the investigated 27,955 authors 12,596 scientists (45.1%) were manually assigned a gender according to their first names. Incomplete scientometric information prevented gender investigations of 14,945 authors (53.5%) as their first names were only available as initials. In 703 cases (<0.1%) names did not permit the drawing of definite conclusions about gender. These 15,359 authors (54.9%) therefore had to be excluded. The obtained data was then combined with the results from the country specific analyses and further investigated. The institutions contributing scientific research to this field were analyzed, evaluated and compared according to their publications and citations. Similarly, subject areas as defined in the original WoS classification as well as scientific journals were analyzed. The volume of scientific cooperation between 1935 and December 2012 was assessed and analyzed. Anamorphous *Density Equalizing Map Projections* (DEMPs) were used for better visualization and understanding of the data [13,14].

## 3. Results and Discussion

### 3.1. Results

#### 3.1.1. Scientific Performance

**Figure 1:**
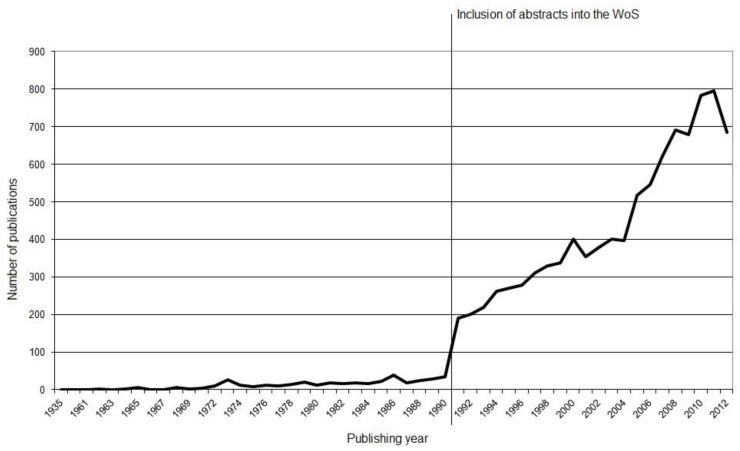
Number of publications.

The analysis of 10,043 publications reveals an overall increase in scientific productivity within the field of *smoking and pregnancy*. Over a time period of 60 years the scientific development remains stable on a low level of not more than 40 annual publications, but sharply increases from 35 to 190 at the beginning of the 1990s with the inclusion of abstracts and key words into the WoS and continues to increase in the present time [15]. The highest number of publications in the investigated time period is achieved in the year 2011 with 795 publications, accounting for 12.6% of the entire scientific production about *smoking and pregnancy*. A similar development is seen with the annual citation rates, which increase at the beginning of the 1990s from only up to 1,000 citations to 7,640 citations. A predominantly continuous growth of the mean bibliography is observed from 15.6 sources in 1985 to 41.8 sources in the present (Figure 1). 

#### 3.1.2. National Analysis

**Figure 2:**
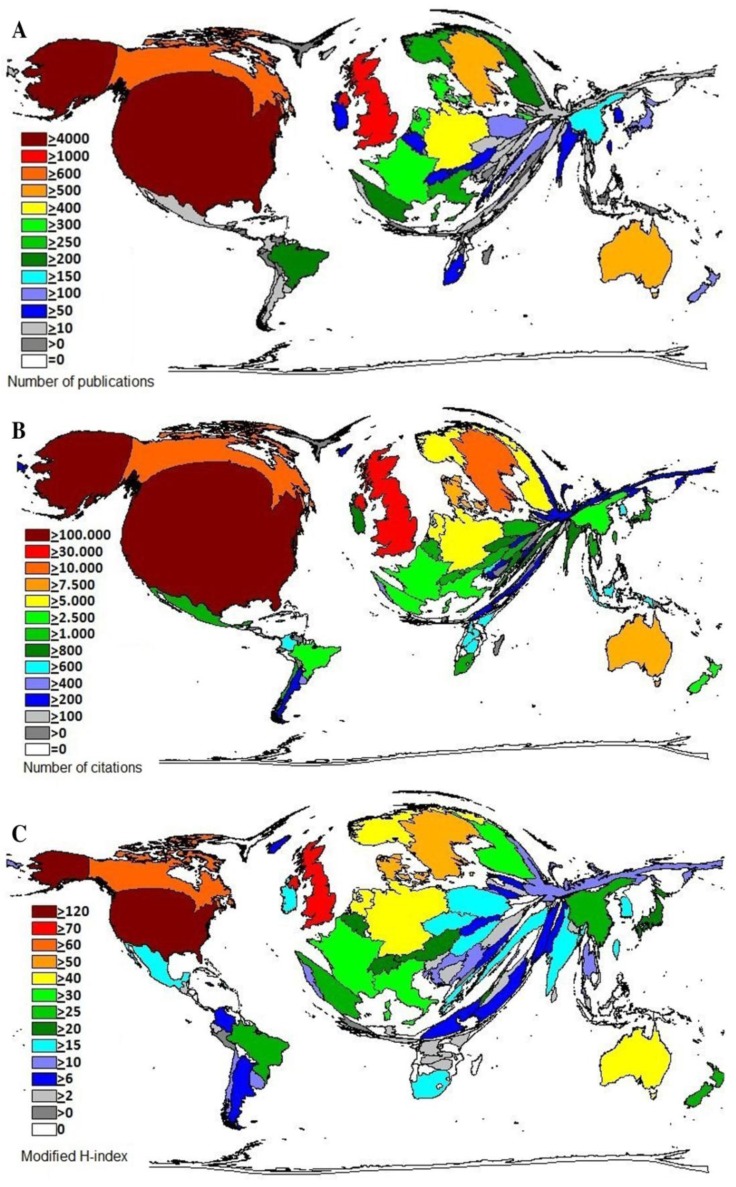
Density equilizing map projections. (**A**) National publication volumes;(**B**) National citation volumes; (**C**) National modified *h-indices*.

The largest number of scientific works about *smoking and pregnancy* is published by the USA with 4,284 (35.5%). The USA is the only nation with more than 4,000 publications (Figure 2A). The nation with the second most published works and the single other country with a four-digit number is the UK with 1,203 works (9.9%). The nation of Canada achieves 634 publications (5.3%). Analyses of national citation volumes and modified *h-indices* show similar results, as the USA achieves the highest total national citation volume with 112,136 citations (41.4%) and a modified *h-index* of 128. At a considerable distance the UK is placed second with a citation volume of 31,651 (8.6%) and a modified *h-index* of 79. The countries Canada (14,427 citations; modified *h-index* 62) and Sweden (13,334 citations, modified *h‑index* 59) on the 3rd and 4th place achieve a citation volume between 10,000 and 30,000 (Figure 2B,C).

**Figure 3:**
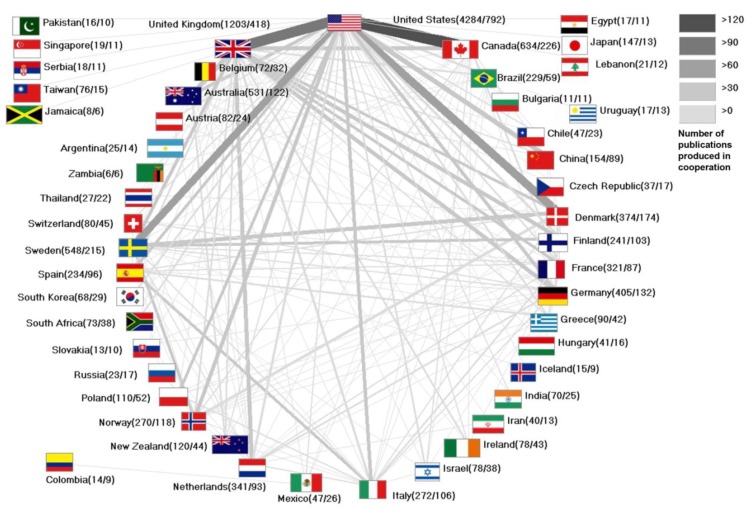
Cooperations of nations.

Note: Numbers in brackets (publication volumes/number of cooperation publications).

The international cooperation on *smoking and pregnancy* is investigated and analyzed (Figure 3). The USA achieves the highest amount of overall international cooperation, as it produces 126 publications together with Canada, 111 scientific works in cooperation with the UK, 85 works with Denmark and 81 publications in cooperation with Sweden. 

#### 3.1.3. Author Analysis

The analysis of the overall publication volume and overall citations reveals that Jorn Olsen and Sven Cnattingius are the most productive scientists within the field of *smoking and pregnancy*. Olsen is the most productive author, with 106 publications and the second most cited scientist with 3,164 citations. He is first-author of 10 scientific works (9.4%) and has the second largest number of senior-authorships with 48 publications. Cnattingius is the second most productive author with 73 publications and the most cited author with 3,321 citations. In the investigated scientific field the achieves the highest absolute number of first-authorships with 18 publications (24.6%). Additionally, he is the senior-author of further 25 works. Gideon Koren has the third most publications about *smoking and pregnancy* and is the 5th most cited researcher with 1,837 citations. He is first-author of 10 publications (14.7%) as well as senior-author of 45 scientific publications. Gary Shaw with 33.3% has the highest relative percentage of first-authorships (13 publications). He is listed as senior-author of a further six publications. The scientist Kypros Nicolaides is listed as senior-author of 49 scientific works (98%). He is the specialist with the highest relative and absolute number of senior-authorships in this field of scientific publishing.

**Figure 4:**
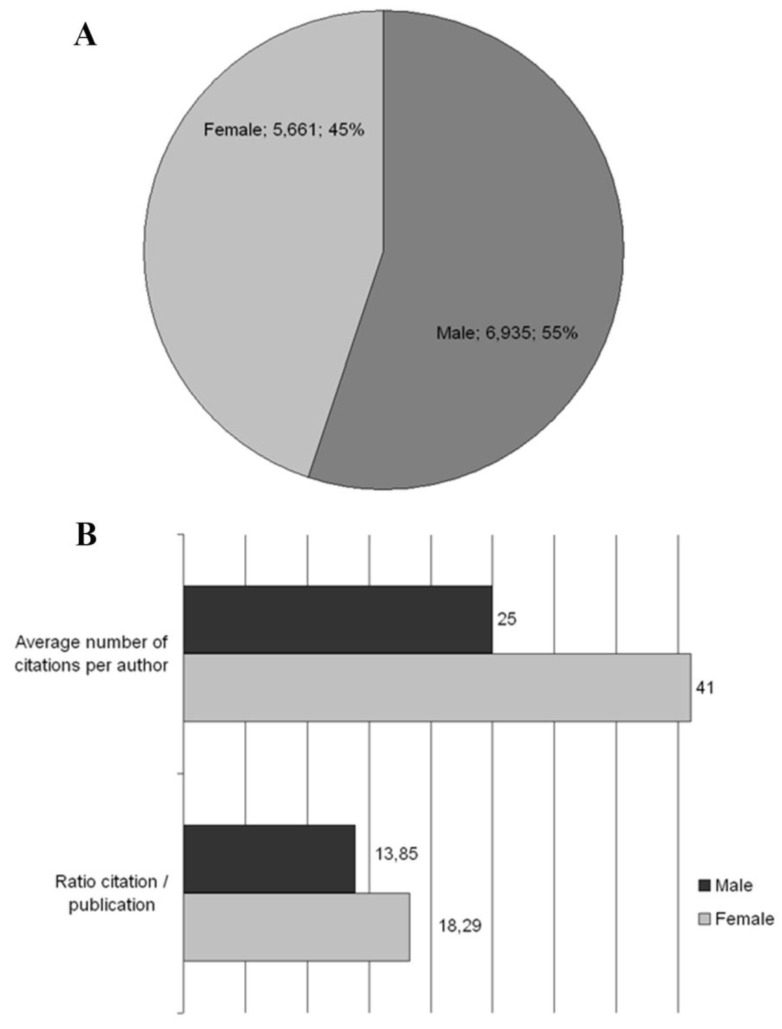
Gender analyses. (**A**) Proportional distribution of the gender of authors; (**B**) Average number of citations per author and ratio citation/publication of the gender of the authors.

The gender analysis includes 12,596 authors contributing to the topic of *smoking and pregnancy*; it reveals that 6,935 scientists (55.1%) are female and 5,661 researchers (44.9%) are male (Figure 4A). Female scientists contribute a total of 12,470 publications (49.7%), which are cited 172,733 times (42.8%). The male scientists reach a publication output of 12,600 works (50.3%) and a citation volume of 230,480 (57.2%). A citation/publication ratio of 18.29 for male authors and a ratio of 13.85 for female authors is calculated. Each female scientist achieves on average 1.8 publications and 25 citations, while her male colleague arrives at an average of 2.2 works and 41 citations (Figure 4B). 

**Figure 5:**
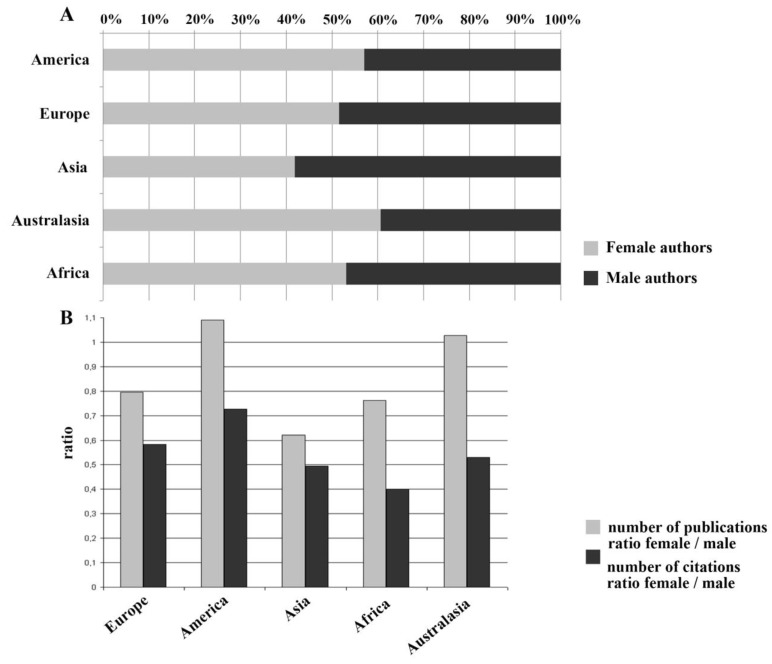
Regional gender analyses. (**A**) Proportion of the number of female and male authors according to continents; (**B**) Female/male publication ratios and female/male citation ratios.

The gender analysis by continents reveals for the continent of America a total of 1,587 female researchers (57.1%) about *smoking and pregnancy* and 1,194 male scientists (42.9%). In Europe 1,266 female authors (51.6%) work together with 1,187 male experts (48.4%). The lowest percentage of female experts is found in Asia, where female authors account for only 41.9%. These 165 female scientists conduct research with 229 male scientists (58.1%). The continent of Australasia enjoys the highest female author percentage with 60.7%. These 213 female researchers work with 138 male scientists (39.3%). In Africa 33 female scientists researching about *smoking and pregnancy* account for 53.2%, while their 29 male colleagues arrive at 46.8% (Figure 5A).

In America female authors have the highest absolute and relative publication volume of 3,881 (52.1%), while their male colleagues produced 3,561 publications (47.9%). In Europe female authors had2,691 publications (44.3%) and male scientists contributed 3,380 publications (55.7%). The lowest relative publication output is found in Asia, where with 447 works female scientists account for only 38.3% of the total publications about *smoking and pregnancy*. Accordingly, male authors in Asia produced 773 works (61.7%). In Australasia female authors achieved a publication output of 459 scientific works (50.7%), while their male colleagues had 447 works (49.3%). In Africa 48 publications (43.2%) were produced by female authors, whereas male scientists contributed 63 publications (56.8%). Female authors in America are cited 59,687 times (42.1%). Their male co-workers enjoy a citation rate of 82,207 (57.9%). In Europe female scientists have 31,705 citations (36.8%), whereas male authors are cited 54,392 times (63.2%).

Female experts about *smoking and pregnancy* have 2,130 citations (33.1%) in Asia. Correspondingly, their male colleagues received 4,303 citations (66.9%). In Australasia female scientists enjoy 4,830 citations (34.6%), while male authors are cited 9,123 times (65.4%). 

In Africa female authors achieve the lowest relative and absolute citation rate with only 414 citations (28.6%), while male scientists in Africa are cited 1,033 times (71.4%). Continent-specific female/male publication ratios as well as female/male citation ratios are shown in Figure 4B; a distinct dominance of male authors is seen, with the only exceptions of the American (1.09) and the Australasian (1.03) female/male publication ratios (Figure 5B).

#### 3.1.4. Institution Analysis

**Figure 6:**
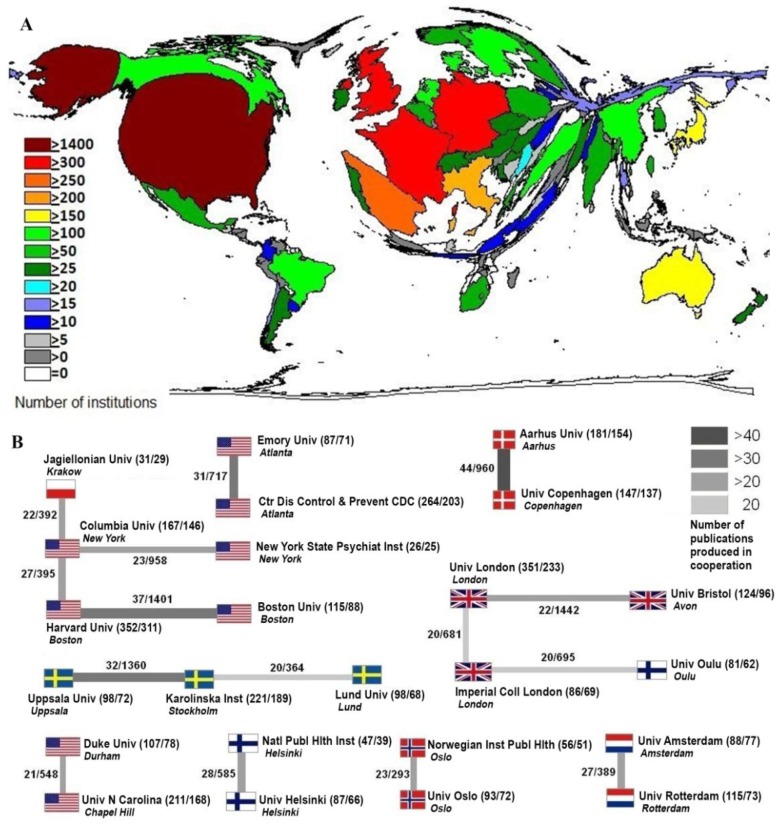
Institution analyses. (**A**) National institution volume; (**B**) Cooperation of institutions.

Notes: numbers in brackets (total amount of publications/the number of publications in cooperation; numbers in between institutions: combined publication volume/combined citations). Univ = University, Coll = College, Inst = Institute, Ctr Dis Control & Prevent = Centers for Disease Control & Prevention, Natl Publ Hlth Inst = National Public Health Institution, New York State Psychiat Inst = New York State Psychiatric Institution, Norwegian Inst Publ Hlth = Norwegian Institute for Public Health, Univ N Carolina = University of North Carolina.

The institutions contributing scientific works to the field of *smoking and pregnancy* are analyzed according to their country of origin (Figure 6A). The highest number of 1,479 institutions is found in the USA. The USA is the only nation with more than 1,400 institutions. The UK possesses the second highest number; 341 British institutions contribute to the worldwide research about *smoking and pregnancy*. Together with France (326) and Germany (301), Spain (255) is the only other country with more than 250 institutions. The analyzed publications about *smoking and pregnancy* are assigned to their respective institutions. The two institutions with the highest scientific output are the Harvard University in Boston, USA with 352 publications and the University of London, UK with 351 publications. They are followed by the Centers for Disease Control and Prevention (CDC) from Atlanta, USA with 264 scientific works. 

Additionally, the cooperation between different institutions is analyzed (Figure 6B). The highest number of cooperation publications on *smoking and pregnancy* is achieved by two Danish institutions, the University of Copenhagen and Aarhus University with 44 publications and 960 citations. Harvard University and Boston University published 37 scientific works about *smoking and pregnancy* and thus achieved the second highest citation rate with 1,401 citations. Two cooperating Swedish institutions, the Uppsala University and the Karolinska Institute produced 32 publications and 1,360 citations. 

#### 3.1.5. Journal Analysis

*The American Journal of Obstetrics & Gynecology* and *The American Journal of Epidemiology* are the most productive scientific journals about the topic of *smoking and pregnancy*. *The American Journal of Obstetrics & Gynecology* publishes 284 works and is cited 9,166 times. This citation rate is exceeded only by the second most productive journal, *The American Journal of Epidemiology*, which publishes 274 works and obtains the highest citation rate of the investigated journals with 9,928 citations. *The American Journal of Epidemiology* achieves a larger average citation index (36.23) in comparison to the more productive *American Journal of Obstetrics & Gynecology*, which possesses an average citation index of 32.27. In addition to being the third most industrious journal with 230 publications, *Obstetrics and Gynecology* maintains the third largest number of citations (8,513) and possesses the 4th largest average citation index (37.01). 

### 3.2. Discussion

#### 3.2.1. Methodical Discussion

*Smoking and pregnancy* is a wide field which is not only limited by the nine months of pregnancy and the smoking of tobacco, but combines a variety of influences and covers topics such as socio-economic questions, different intercultural factors, globalization and shifting paradigms as well as long-term effects over more than one generation [9,16]. The basis for the data used in this investigation is the WoS, which is one of the largest indexed interdisciplinary scientific online databases [15]. 

The scientometric analyses used in this study were aimed at investigating two important aspects of science: quality and quantity of the global available data on the field of *smoking and pregnancy*. The scientific value can be broken down into the two main elements of quantity (*i.e.*, productivity) and quality (*i.e.*, citation rate), both of which can be measured to a large degree by the help of scientometric tools. Even if the following discussion identifies the strong and weak points of the used scientometric techniques, the numerous benefits of an objective scientometric investigation about *smoking and pregnancy* cannot be denied.

In this study a connection is established between the quality of a scientific publication and the quantity of its citation based on data obtained using the WoS function *citation report*, generally assuming that a large citation rate and therefore a large scientific resonance is an indicator of scientific quality. The obtained number of citations was interpreted as a criterion for the amount of publicity and resonance a publication obtained. This functioned as a marker for the usefulness and importance of a publication in its respective field of science. As a general rule, the higher a publication’s citation rate, the higher its importance for science; however, the phenomenon of self-citation can suggest an inaccurately elevated level of significance [17]. Furthermore, the scientific importance of authors and journals of different scientific fields cannot be directly compared to each other.

#### 3.2.2. Content-related Discussion

Analyzing the data on international scientific productivity a scientific dominance of the USA, the UK and Canada is observed. The USA has a publication power of 4,284 scientific works (35.5%) and a national volume of 112,136 citations (41.4%). The UK produced 1,203 publications (9.9%) and 31,651 citations (8.6%), whereas Canada arrives at 634 scientific works (5.3%) and 14,427 citations (5.3%). Together these three leading nations contribute 50.7 % of the total publications which account for 55.3% of the overall citations. This is due to different reasons; firstly, these developed countries enjoy a large number of well established scientific research facilities. A large and developed country like the USA can maintain a large number of modern and well equipped research facilities which generate a significantly higher scientific output compared to less developed countries with fewer scientific facilities [18]. Secondly, these countries enjoy a high level of general public education and are well known for their scientific traditions. Therefore they attract more foreign scientists who are eager to perform research in internationally well known scientific facilities. This phenomenon is explained by the *Matthew Effect* [18,19]. In these countries the public and economic interest about health is most prominent, which plays an important role in further promoting and facilitating research for prophylaxis and therapies. Considering these factors, it is likely that the USA, the UK and Canada will continue to be the predominant nations with the highest scientific importance about *smoking and pregnancy*. With rapidly increasing globalization and development it is to be expected that the global scientific output in this field further increases in the future [18,20].

The qualities of national scientific works are measured by national citation rates. Without the inclusion of a threshold of a minimum of 30 publications several countries would reach exceptionally high national citation rates. Nations like Zimbabwe reach citation rates up to 155, exceeding well established countries like the USA, UK or Canada. Seychelles contributed only one publication about *smoking and pregnancy*, but it was cited 54 times. This points out a distinct weakness of the national citation rate and illustrates why a threshold of 30 publications was chosen. Because of the very low number of publications of some countries in combination with the strong preference of scientists to cite national colleagues a large bias is created. Because of these shortcomings the analysis of the modified h-index is performed, resulting in a completely different representation where the USA, the UK and Canada are positioned in the first places. To appropriately evaluate the quality of scientific work a combination of different scientometric instruments is needed.

Among 27,955 authors contributing to *smoking and pregnancy* only 292 authors (1.0%) achieve more than 10 publications and are regarded as the most productive experts. The authors Jorn Olsen, Sven Cnattingius, Gideon Koren and Kypros Nicolaides are identified as the leading experts about smoking and pregnancy. They benefit from their long careers in the scientific community and their reputation, which explains the large percentage of their senior-authorships. Assuming that the first- and senior-authorships are the more important and responsible positions, the role of the co-author has to be scrutinized carefully. Especially with an increasing number of co-authors the significance of the scientific contribution of each participant cannot be satisfactorily evaluated. 

If the threshold of 15 publications had not been applied, several authors with a low publication output about this topic would have been included in the results; however, for productive authors the modified *h-index* is a reliable indicator of resonance and scientific importance. 

Out of the total number of 27,955 authors contributing to *smoking and pregnancy* 12,596 authors (45.1%) can be assigned a specific gender, resulting in the assignment of 25,070 publications and 403,213 citations to either a female or male author. Even though in 703 cases (<0.1%) names did not permit drawing of definite conclusion about gender and incomplete scientometric information prevented gender investigations of 14,945 authors (53.5%) as their first names were only available as initials, assuming that high bibliographic quality and completeness of scientometric information is not biased by gender or country the investigated figures constitute a general representation and allow conclusions about the entire research field of *smoking and pregnancy*. 

Male scientists tend to publish more works compared to their female co-workers. Additionally, they are cited almost twice as often as their female colleagues (41 citations *vs.* 25 citations). These numbers can be explained by several factors. Firstly, medicine itself is becoming more female driven; in the year 2012 in Germany 64% of medicine students were women. Secondly, female doctors tend to choose certain specialties. In Germany, the specialty of gynecology and obstetrics currently has more than 90% female doctors and approximately 75% female doctors are employed in the field of pediatrics [21,22]. These are two of the three main subject areas publishing about *smoking and pregnancy*. This relatively young and inexperienced new generation of female doctors has already achieved a certain number of publications and is therefore included in this scientometric investigation. However, for female researchers it is more difficult to organize the numerous demands of an academic career in combination with a family. The most demanding times of scientists’ lives are their 20s and 30s when the foundation for their careers is laid. For female scientists this corresponds to the period in life when women traditionally start a family, whereas male scientists can postpone having children longer. As women traditionally handle most of the work related to family and children, male authors are less restricted and have more time to be scientifically productive [23,24,25]. The previous generation of medical scientists is still mainly composed of male doctors. Because of their scientific experience and their high reputation they are more productive and are cited more often [26,27]. 

A total of 6,041 scientists can be further linked to a specific country and analyzed according to continents. The highest percentage of female authors about *smoking and pregnancy* is found in the modern western nations Australia and New Zealand, where women have achieved a high level of gender equality in the past [28]. The lowest percentage of female scientists is present in Asia, where the traditions and the high workload might hinder female scientists from being more productive. In America female authors achieve the highest relative publication volume. The lowest relative female publication output is found in Asia. This is explained by the dominance of the western nations of the USA and Canada. Within the total gender-assignable American publications (7,442) these two countries together account for 7,004 scientific works (94.1%). A comparatively high level of gender equality is implemented in these Northern American societies, leading to evenly balanced female production. Central- and South-American countries with a lower level of gender equality are less important due to their low percentage of publications. For the above mentioned reasons the opposite is true for the continent of Asia [18]. The highest percentage of female citations is achieved by authors in America, whereas in Africa female authors achieve the lowest relative citation rate. The USA and Canada account for 138,932 out of 141,894 American citations (97.9%). In Africa the more traditional role of women is likely to be the reason for their low citation rates and resonances.

A total of 5,471 institutions participate in research about *smoking and pregnancy*. The majority of the institutions (1,479) are located in the USA, accounting for 27.0% of the global research facilities. The second highest number of institutions is located in the UK with 341 research facilities (6.2%), followed by France with 326 institutions (6.0%). The most productive institutions about *smoking and pregnancy* are two of the world’s famous and renowned universities, Harvard University in Boston, USA with 352 publications and the University of London, UK with 351 publications. The third most productive institution is the Centers for Disease Control and Prevention located in Atlanta, USA with 264 scientific works. These three most productive institutions also gain the most attention within the scientific community of *smoking and pregnancy*, as they achieve the highest citation rates of 12,844; 11,755 and 7,650 citations, in that order. Together they achieve 11.9% of the total citations. Harvard University employs several of the most productive scientists about smoking and pregnancy like Joseph Biederman (1,459 citations), whereas the leading expert Kypros Nicolaides (1,111 citations) works at the University of London. The Centers for Disease Control and Prevention is a U.S. federal agency with the aim to observe and promote public health, research and implement prevention strategies and create safe and healthful environments [29].

## 4. Conclusions

In the future medicine and scientific research will become increasingly specialized while the quality of scientific work in a global community will increase further. As a result research will become even more expensive, requiring more international communication and cooperation. The combination of these facts leads to a prognosis of an increase in international communication about *smoking and pregnancy* in the future. More than half of the scientists contributing to this field are female, nevertheless they achieve less than half of the publications. Additionally, only 42.8% of the citations are accomplished by female authors. The highest percentage of these female experts is found in Australasia, while the lowest percentage of female scientists is positioned in Asia. A slow improvement towards more gender equality within this scientific field is to be expected in the future. Furthermore, the continuous increase in quality and quantity of scientific research about *smoking and pregnancy* is a reliable indicator for the increasing dynamics of this subject, proving the growing development of research and a higher public interest. This scientometric and gender analysis presents an interim assessment of the scientific development and provides a contribution to the ongoing mission to understand, prevent and fight smoking and pregnancy.
